# Postbuckling Investigations of Piezoelectric Microdevices Considering Damage Effects

**DOI:** 10.3390/s140304876

**Published:** 2014-03-11

**Authors:** Zhigang Sun, Xianqiao Wang

**Affiliations:** 1 College of Energy and Power Engineering and State Key Laboratory of Mechanics and Control of Mechanical Structures, Nanjing University of Aeronautics and Astronautics, Nanjing 210016, China; 2 College of Engineering, University of Georgia, Athens, GA 30602, USA

**Keywords:** piezoelectric plates, tensor valued internal state variables, postbuckling behavior, damage evolution, initial deflections

## Abstract

Piezoelectric material has been emerging as a popular building block in MEMS devices owing to its unique mechanical and electrical material properties. However, the reliability of MEMS devices under buckling deformation environments remains elusive and needs to be further explored. Based on the Talreja's tensor valued internal state damage variables as well as the Helmhotlz free energy of piezoelectric material, a constitutive model of piezoelectric materials with damage is presented. The Kachanvo damage evolution law under in-plane compressive loads is employed. The model is applied to the specific case of the postbuckling analysis of the piezoelectric plate with damage. Then, adopting von Karman's plate theory, the nonlinear governing equations of the piezoelectric plates with initial geometric deflection including damage effects under in-plane compressive loads are established. By using the finite difference method and the Newmark scheme, the damage evolution for damage accumulation is developed and the finite difference procedure for postbuckling equilibrium path is simultaneously employed. Numerical results show the postbuckling behaviors of initial flat and deflected piezoelectric plates with damage or no damage under different sets of electrical loading conditions. The effects of applied voltage, aspect ratio of plate, thick-span ratio of plate, damage as well as initial geometric deflections on the postbuckling behaviors of the piezoelectric plate are discussed.

## 1. Introduction

The use of piezoelectric materials in intelligent structures has received considerable attention in recent years due to the intrinsic direct and converse piezoelectric effects. Piezoelectric materials have been used as sensors or actuators for the control of the active shape or vibration of structures. Defects such as microcracks, voids, dislocations and delamination are introduced in piezoelectric materials during the manufacturing and poling process. The existence of these defects greatly affects the electric, dielectric, elastic, mechanical and piezoelectric properties of the piezoelectric materials, especially the service life of piezoelectric structures. When subjected to mechanical and electrical loads, these defects may grow in size and cracks may propagate leading to premature mechanical or electrical fatigue failure. Therefore, it is important to understand the growth of these defects, the damage accumulation and the overall effect of these defects on the average mechanical and electrical properties of piezoelectric structures.

Damage in fiber-reinforced composite materials has been extensively investigated, and many theories have been established and used to predict the life of composite structures. Based on the framework of irreversible thermodynamics with internal state variables, Talreja [1] developed a phenomenological theory for composite laminated plates. In his study, the Helmholtz free energy was expanded into a polynomial in terms of elastic strains and damage variables to obtain the stiffness-damage relations. Utilizing a continuum mechanics approach, Allen *et al.* [2,3] developed a model for predicting the thermomechanical constitution of initially elastic composites subjected to both monotonic and cyclic fatigue loading. Valliappan *et al.* [4] established the elastic constitutive equations for anisotropic damage mechanics, and the implementation of these constitutive equations in the finite element analysis was explained. By defining damage variables as the material stiffness reduction, Ladeveze and Dantec [5] formulated the constitutive equations and the corresponding damage evolution laws of the elementary ply for laminated composites that can be used to describe the matrix micro-cracking and fiber/matrix debonding. Schapery and Sicking [6] discussed the homogenized constitutive equations for the mechanical behavior of unidirectional fiber composites with growing damage, and the emphasis was on resin matrices reinforced with high modulus elastic fiber. Zhang *et al.* [7] investigated a computational model for the damage evolution of engineering materials under dynamic loading, and two models for dynamic damage evolution of materials in general anisotropic damage state were presented. Moore and Dillard [8] have observed time dependent growth of transverse cracks in graphite/epoxy and Kevlar/epoxy cross-ply laminated at room temperature. Luo and Daniel [9] have shown that the macroscopic mechanical behavior of unidirectional fiber-reinforced brittle matrix composites can be correlated explicitly with the microscopic deformation and damage.

Modeling and analysis of multilayer piezoelectric beams and plates have reached a relative maturity as attested by the numerous papers. Mindlin [10] presented the theory of piezoelectric crystals plate considering shear and bending. Chandrashekhara and Tiersten [11] developed general piezoelectric nonlinear theory and detailed the vibration equations of different piezoelectric crystals. Chandrashkhara, Tenneti [12] and Zhou *et al.* [13] investigated the dynamic control of laminated piezoelectric plates by the finite element (FE) method. Wang and Rogers [14] presented a model for laminated plates with spatially distributed piezoelectric patches. Tzou and Gadre [15] analyzed thin laminates coupled with shell actuators for distributed vibration control. Xu *et al.* [16] analyzed the free vibration of laminated piezothermoelectric plate based on the 3D theory. Mitchell and Reddy [17] proposed the theory of the laminated piezoelectric plates by using classical plate theory and simple third-order theory, respectively. Noor and Peters [18] presented postbuckling analysis of multilayered composite plates subjected to combined axial and thermal loads and investigated the effects of mechanical and thermal loading on postbuckling behaviors of composite plates. Based on transverse shear-deformable theory, Librescu and Souza [19] gave the postbuckling analysis of geometrically imperfect flat panels under combined thermal and compressive edge loadings. Shen [20] investigated the postbuckling behaviors of laminated plates with piezoelectric actuators under complex loading conditions based on Reddy's higher order shear deformation plate theory. Oh *et al.* [21] studied thermal postbuckling behavior of laminated plates with top and/or bottom actuators subjected to thermal and electrical loads. In their analysis all the static/dynamic behaviors of laminated plates were investigated without considering the damage effects which would greatly influence the mechanical behaviors of smart structures. To the best of the author's knowledge, up to now postbuckling analysis of piezoelectric structures considering the damage effects has rarely been investigated and reported. For example, Aydin [22] studied the dynamic characteristics of functionally graded beams with open edge cracks, in which an analytical method was proposed to determine the free vibration of beams with any number of cracks. Mao *et al.* [23] studied the creep buckling and post-buckling of laminated piezoelectric viscoelastic functionally graded material (FGM) plates by adopting the Boltzmann superposition principle, and the nonlinear creep buckling governing equations of the laminated piezoelectric viscoelastic FGM plates with initial deflection were derived on the basis of the Reddy's higher-order shear deformation plate theory. Hamed [24] presented a nonlinear theoretical model for their bending and creep buckling analysis. The model accounted for the viscoelasticity of the materials using differential-type constitutive relations that were based on the linear Boltzmann's principle of superposition. Xu *et al.* [25] presented a high performance and simple structure bi-stable piezoelectric energy harvester based on simply supported piezoelectric buckled beam. Cottone *et al.* [26] investigated an approach for piezoelectric beams by exerting an increasing axial compression and demonstrated that the numerical model and experimental results were in good qualitative agreement. A constitutive model of fully coupled electro-magneto-thermo-elastic multiphase composites has been proposed by Aboudi [27]. In his works, the linear displacement, electric potential and magnetic potential are adopted, which can't predict the micro fields precisely. Bansal and Pindera [28] proposed a unified macro-and micro-mechanics failure model with method of cells as finite-volume direct averaging micromechanics (FVDAM). Based on the FVDAM theory, Sun *et al.* [29] built a unified macro- and micro-mechanics constitutive model of fully coupled fields in composite materials by high-order displacement, electric potential and magnetic potential.

In the present study, a new constitutive model for piezoelectric materials using the Talreja's tensor valued internal state damage variables and the Helmhotlz free energy of piezoelectric material is presented. This model is then applied to a specific case of postbuckling analysis of piezoelectric plates under in-plane compressive loads. By adopting von Karman's plate theory and using the finite difference and the Newmark scheme, the damage evolution for damage accumulation is developed and the finite difference procedure for postbuckling equilibrium path is simultaneously employed. In the numerical examples, the effects of variation in the load parameters, damage influences and geometric parameters of the plate on postbuckling equilibrium paths are discussed.

## 2. Basic Equations

### 2.1. Constitutive Equations for Damaged Piezoelectric Materials

**Figure 1.**
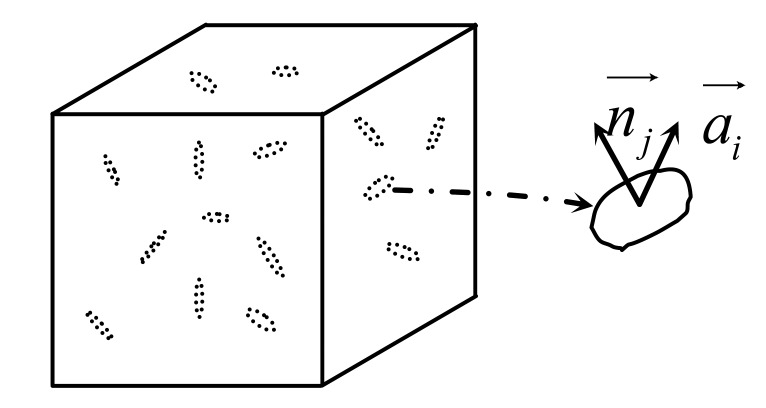
Representative volume element with internal damage variables for piezoelectric materials.

Consider a representative volume element of a piezoelectric solid with a multitude of damage entities in the form of microcracks, as shown in Figure 1. As discussed in Talreja, two vectors are needed to define each damage entity. These are the damage influence vector $\vec{a_{i}}$ and the $\vec{n_{i}}$ unit normal to the damage entity surface. The damage influence vector represents an appropriately chosen effect of the damage entity on the surrounding medium. With these two vectors, a damage entity tensor *d_ij_* is formed by taking an integral of the diad $\vec{a_{i}}\vec{n_{j}}$ over the surface of the damaged entity:

(1) $$d_{ij}=\int_{S}\vec{a_{i}}\vec{n_{j}}dS$$

where *S* is the damage entity surface.

Now if there are *n* distinct damage modes in the representative volume element (e.g., intralaminar cracks in different orientations, *etc*.) denoted by *k* = 1,2,…*n*, a damage tensor can be defined for each mode as:

(2) $$\omega_{ij}^{k}=\frac{1}{V_{r}}\underset{\vartheta_{k}}{\text{∑}}{\left(d_{ij}\right)}_{\vartheta_{k}}$$

where *V_r_* is the volume of the representative volume element and *ϑ_k_* represents the number of damage entities in the *k*th damage mode. The tensor $\omega_{ij}^{k}$ is an unsymmetrical tensor in general. However, we can represent the vector $\vec{a_{i}}$ along the normal and tangential directions at any point on the surface of the damage entity and write:

(3) $$d_{ij}=d_{ij}^{1}+d_{ij}^{2}$$

where $d_{ij}^{1}=\int_{S}a\vec{n_{i}}\vec{n_{j}}dS$ and $d_{ij}^{2}=\int_{S}b\vec{m_{i}}\vec{n_{j}}dS$. in which, *a* and *b* are the magnitudes of the normal and tangential projections of vector $\vec{a_{i}}$ respectively, and vectors $\vec{n_{i}}$ and $\vec{m_{i}}$ are unit normal and tangential vectors, respectively. Thus the damage tensor *ω_ij_* can be written as:

(4) $$\omega_{ij}^{k}=\omega_{ij}^{1k}+\omega_{ij}^{2k}$$

where $\omega_{ij}^{1k}=\underset{\vartheta_{k}}{\text{∑}}{\left(d_{ij}^{1}\right)}_{\vartheta_{k}}/V_{r}$ and $\omega_{ij}^{2k}=\underset{\vartheta_{k}}{\text{∑}}{\left(d_{ij}^{2}\right)}_{\vartheta_{k}}/V_{r}$.

Physically, the damage tensor $\omega_{ij}^{1k}$ represents the effects of crack opening on the surrounding medium whereas the damage tensor $\omega_{ij}^{2k}$ represents the effects of sliding between the two crack faces. In many situations, the sliding between the crack faces can be negligible, e.g., for intralaminar cracks constrained by stiff plies, and hence we assume $\omega_{ij}^{2k}\equiv 0$. This implies $\omega_{ij}^{k}\equiv \omega_{ij}^{1k}$ which is a symmetric tensor.

For the case of damaged piezoelectric material without temperature effect where the damage is represented by internal state variables, the Helmholtz free energy of piezoelectric material can be written as a function of the transformed elastic strains, the electric field vector and damage internal variables, that is:

(5) $$H=H(\varepsilon_{ij},E_{i},\omega_{ij}^{k})$$

The transformed stress components *σ_ij_* and the electric displacement components *G_i_* at any fixed damage state are now given by:

(6) $$\begin{array}{ll} \sigma_{ij} & =\frac{\partial H(\varepsilon_{ij},E_{i},\omega_{ij}^{k})}{\partial \varepsilon_{ij}}\\ G_{i} & =-\frac{\partial H(\varepsilon_{ij},E_{i},\omega_{ij}^{k})}{\partial E_{i}} \end{array}$$

When the damage induced by the cracks in the piezoelectric material has the orthotropic property, the irreducible integrity bases for a scalar polynomial function of two symmetric second rank tensors can be expressed as [30]:

(7) $$\begin{array}{l} \varepsilon_{11},\varepsilon_{22},\varepsilon_{33},{\varepsilon_{23}}^{2},{\varepsilon_{31}}^{2},{\varepsilon_{12}}^{2},\varepsilon_{12}\varepsilon_{23}\varepsilon_{31},\\ \omega_{11}^{k},\omega_{22}^{k},\omega_{33}^{k},{\left(\omega_{23}^{k}\right)}^{2},{\left(\omega_{31}^{k}\right)}^{2},{\left(\omega_{12}^{k}\right)}^{2},\omega_{12}^{k}\omega_{23}^{k}\omega_{31}^{k}\\ \begin{array}{ll} \varepsilon_{23}\omega_{23}^{k},\varepsilon_{31}\omega_{31}^{k},\varepsilon_{12}\omega_{12}^{k},\omega_{23}^{k}\varepsilon_{12}\varepsilon_{13},\omega_{31}^{k}\varepsilon_{32}\varepsilon_{12},\omega_{12}^{k}\varepsilon_{13}\varepsilon_{23}, & \left(k=1,2,\cdot \cdots \cdots n\right) \end{array}\\ \varepsilon_{23}\omega_{12}^{k}\omega_{13}^{k},\varepsilon_{31}\omega_{32}^{k}\omega_{12}^{k},\varepsilon_{12}\omega_{13}^{k}\omega_{23}^{k}\\ E_{1},E_{2},E_{3} \end{array}$$

where *n* is the number of the cracks' direction in the material. For a piezoelectric single-layer plate, the local coordinate system *o* −1−2−3 is selected, in which 1,2 denote the two principal direction of the piezoelectric plate, 3 is vertical to the midsurface. According to the Kirchhoff hypothesis for plate *ε*_13_ = *ε*_23_ =0 and applying Voigt notation to describe strains and damage variables, the bases of invariants can be further written as:

(8) $$\begin{array}{cc} \begin{array}{l} \varepsilon_{1},\varepsilon_{2},\varepsilon_{3},{\varepsilon_{6}}^{2},\omega_{1}^{k},\omega_{2}^{k},\omega_{3}^{k},{\left(\omega_{4}^{k}\right)}^{2},{\left(\omega_{5}^{k}\right)}^{2},{\left(\omega_{6}^{k}\right)}^{2},\\ \omega_{4}^{k}\omega_{5}^{k}\omega_{6}^{k},\varepsilon_{6}\omega_{6}^{k},\varepsilon_{6}\omega_{4}^{k}\omega_{5}^{k}\\ E_{1},E_{2},E_{3} \end{array} & ,(k=1,2,\cdot \cdots \cdots n) \end{array}$$

Using the above stated irreducible integrity bases, the Helmholtz free energy of piezoelectric materials can be expressed as a quadratic expression of the strains or the electric field intensity, a mixture quadratic expression of strains and electric field intensity and a linear expression of damage variables [31] as follows:

(9) $$\begin{array}{ll} H & =C_{1}^{0}\varepsilon_{1}^{2}+C_{2}^{0}\varepsilon_{1}\varepsilon_{2}+C_{3}^{0}\varepsilon_{2}^{2}+C_{4}^{0}\varepsilon_{6}^{2}+C_{5}^{0}\varepsilon_{1}^{2}+C_{6}^{0}\varepsilon_{1}\varepsilon_{3}+C_{7}^{0}\varepsilon_{2}\varepsilon_{3}-(\kappa_{1}^{0}E_{1}^{2}+\kappa_{2}^{0}E_{2}^{2}+\\ & \kappa_{3}^{0}E_{3}^{2}+\kappa_{4}^{0}E_{1}E_{2}+\kappa_{5}^{0}E_{2}E_{3}+\kappa_{6}^{0}E_{3}E_{1})-(e_{1}^{0}E_{1}\varepsilon_{1}+e_{2}^{0}E_{1}\varepsilon_{2}+e_{3}^{0}E_{1}\varepsilon_{3}+e_{4}^{0}E_{2}\varepsilon_{1}+\\ & e_{5}^{0}E_{2}\varepsilon_{2}+e_{6}^{0}E_{2}\varepsilon_{3}+e_{7}^{0}E_{3}\varepsilon_{1}+e_{8}^{0}E_{3}\varepsilon_{2}+e_{9}^{0}E_{3}\varepsilon_{3})+\overset{n}{\underset{k=1}{\text{∑}}}(C_{1}^{k}\varepsilon_{1}^{2}\omega_{1}^{k}+C_{2}^{k}\varepsilon_{1}^{2}\omega_{2}^{k}+C_{3}^{k}\varepsilon_{1}^{2}\omega_{3}^{k}\\ & +C_{4}^{k}\varepsilon_{2}^{2}\omega_{1}^{k}+C_{5}^{k}\varepsilon_{2}^{2}\omega_{2}^{k}+C_{6}^{k}\varepsilon_{2}^{2}\omega_{3}^{k}+C_{7}^{k}\varepsilon_{3}^{2}\omega_{1}^{k}+C_{8}^{k}\varepsilon_{3}^{2}\omega_{2}^{k}+C_{9}^{k}\varepsilon_{3}^{2}\omega_{3}^{k}+C_{10}^{k}\varepsilon_{6}^{2}\omega_{1}^{k}+\\ & C_{11}^{k}\varepsilon_{6}^{2}\omega_{2}^{k}+C_{12}^{k}\varepsilon_{6}^{2}\omega_{3}^{k}+C_{13}^{k}\varepsilon_{1}\varepsilon_{2}\omega_{1}^{k}+C_{14}^{k}\varepsilon_{1}\varepsilon_{2}\omega_{2}^{k}+C_{15}^{k}\varepsilon_{1}\varepsilon_{2}\omega_{3}^{k}+C_{16}^{k}\varepsilon_{2}\varepsilon_{3}\omega_{1}^{k}+C_{17}^{k}\varepsilon_{2}\varepsilon_{3}\omega_{2}^{k}\\ & +C_{18}^{k}\varepsilon_{2}\varepsilon_{3}\omega_{3}^{k}+C_{19}^{k}\varepsilon_{3}\varepsilon_{1}\omega_{1}^{k}+C_{20}^{k}\varepsilon_{3}\varepsilon_{1}\omega_{2}^{k}+C_{21}^{k}\varepsilon_{3}\varepsilon_{1}\omega_{3}^{k}+C_{22}^{k}\varepsilon_{6}\varepsilon_{1}\omega_{6}^{k}+C_{23}^{k}\varepsilon_{6}\varepsilon_{2}\omega_{6}^{k}+\\ & C_{24}^{k}\varepsilon_{6}\varepsilon_{3}\omega_{6}^{k})-\overset{n}{\underset{k=1}{\text{∑}}}(\kappa_{1}^{k}E_{1}^{2}\omega_{1}^{k}+\kappa_{2}^{k}E_{1}^{2}\omega_{2}^{k}+\kappa_{3}^{k}E_{1}^{2}\omega_{3}^{k}+\kappa_{4}^{k}E_{2}^{2}\omega_{1}^{k}+\kappa_{5}^{k}E_{2}^{2}\omega_{2}^{k}+\kappa_{6}^{k}E_{3}^{2}\omega_{3}^{k}+\\ & \kappa_{7}^{k}E_{3}^{2}\omega_{1}^{k}+\kappa_{8}^{k}E_{3}^{2}\omega_{2}^{k}+\kappa_{9}^{k}E_{3}^{2}\omega_{3}^{k}+\kappa_{10}^{k}E_{1}E_{2}\omega_{1}^{k}+\kappa_{11}^{k}E_{1}E_{2}\omega_{2}^{k}+\kappa_{12}^{k}E_{1}E_{2}\omega_{3}^{k}+\kappa_{13}^{k}E_{2}E_{3}\omega_{1}^{k}\\ & +\kappa_{14}^{k}E_{2}E_{3}\omega_{2}^{k}+\kappa_{15}^{k}E_{2}E_{3}\omega_{3}^{k}+\kappa_{16}^{k}E_{3}E_{1}\omega_{1}^{k}+\kappa_{17}^{k}E_{3}E_{1}\omega_{2}^{k}+\kappa_{18}^{k}E_{3}E_{1}\omega_{3}^{k})-\overset{n}{\underset{k=1}{\text{∑}}}(e_{1}^{k}E_{1}\varepsilon_{1}\omega_{1}^{k}\\ & +e_{2}^{k}E_{1}\varepsilon_{1}\omega_{2}^{k}+e_{3}^{k}E_{1}\varepsilon_{1}\omega_{3}^{k}+e_{4}^{k}E_{1}\varepsilon_{2}\omega_{1}^{k}+e_{5}^{k}E_{1}\varepsilon_{2}\omega_{2}^{k}+e_{6}^{k}E_{1}\varepsilon_{2}\omega_{3}^{k}+e_{7}^{k}E_{1}\varepsilon_{3}\omega_{1}^{k}+e_{8}^{k}E_{1}\varepsilon_{3}\omega_{2}^{k}\\ & +e_{9}^{k}E_{1}\varepsilon_{3}\omega_{3}^{k}+e_{10}^{k}E_{2}\varepsilon_{1}\omega_{1}^{k}+e_{11}^{k}E_{2}\varepsilon_{1}\omega_{2}^{k}+e_{12}^{k}E_{2}\varepsilon_{1}\omega_{3}^{k}+e_{13}^{k}E_{2}\varepsilon_{2}\omega_{1}^{k}+e_{14}^{k}E_{2}\varepsilon_{2}\omega_{2}^{k}+e_{15}^{k}E_{2}\varepsilon_{2}\omega_{3}^{k}\\ & +e_{16}^{k}E_{2}\varepsilon_{3}\omega_{1}^{k}+e_{17}^{k}E_{2}\varepsilon_{3}\omega_{2}^{k}+e_{18}^{k}E_{2}\varepsilon_{3}\omega_{3}^{k}+e_{19}^{k}E_{3}\varepsilon_{1}\omega_{1}^{k}+e_{20}^{k}E_{3}\varepsilon_{1}\omega_{2}^{k}+e_{21}^{k}E_{3}\varepsilon_{1}\omega_{3}^{k}+e_{22}^{k}E_{3}\varepsilon_{2}\omega_{1}^{k}\\ & +e_{23}^{k}E_{3}\varepsilon_{2}\omega_{2}^{k}+e_{24}^{k}E_{3}\varepsilon_{2}\omega_{3}^{k}+e_{25}^{k}E_{3}\varepsilon_{3}\omega_{1}^{k}+e_{26}^{k}E_{3}\varepsilon_{3}\omega_{2}^{k}+e_{27}^{k}E_{3}\varepsilon_{3}\omega_{3}^{k}+e_{28}^{k}E_{1}\varepsilon_{6}\omega_{6}^{k}+e_{29}^{k}E_{2}\varepsilon_{6}\omega_{6}^{k}\\ & +e_{30}^{k}E_{3}\varepsilon_{6}\omega_{6}^{k})+P_{0}+P_{1}\left(\varepsilon_{p},\omega_{q}^{k}\right)+P_{2}\left(\omega_{q}^{k}\right)+P_{3}\left(E_{i},\omega_{q}^{k}\right) \end{array}$$

where $C_{i}^{0}(i=1,2,\cdots 7)$ are the elastic material constants without damage, $e_{i}^{0}(i=1,2,\cdots 9)$ are the piezoelectric constants without damage, $\kappa_{i}^{0}(i=1,2,\cdots 6)$ are the permittivity matrix constants without damage, $C_{i}^{k}(i=1,2,\cdots 24)$ are the material constants with damage; $e_{i}^{k}(i=1,2,\cdots 30)$ are the piezoelectric constants with damage, $\kappa_{i}^{k}(i=1,2,\cdots 18)$ are the permittivity matrix constants with damage, *ρ* is the density of piezoelectric material, *P*_0_ is a constant, *P*_1_ is a linear function of strains, *P*_2_ is a linear function of damage variables and *P*_3_ is a linear function of the electric field intensity. Then the stresses and the electric displacements can be expressed as:

(10) $$\begin{array}{ll} \sigma_{p} & =\frac{\partial H}{\partial \varepsilon_{p}}=[C_{pq}^{0}+\overset{n}{\underset{k=1}{\text{∑}}}C_{pq}^{k}]\varepsilon_{q}-[e_{pm}^{0}+\overset{n}{\underset{k=1}{\text{∑}}}e_{pm}^{k}]E_{m}\\ G_{m} & =-\frac{\partial H}{\partial E_{m}}={\left[e_{qm}^{0}+\overset{n}{\underset{k=1}{\text{∑}}}e_{qm}^{k}\right]}^{T}\varepsilon_{q}+[\kappa_{mn}^{0}+\overset{n}{\underset{k=1}{\text{∑}}}\kappa_{mn}^{k}]E_{n} \end{array}$$

where $C_{pq}^{0}$, $C_{pq}^{k}$, $\kappa_{mn}^{0}$ and $\kappa_{mn}^{k}$ are all symmetric matrixes having the forms as follows:

(11) $$[C_{pq}^{0}]=\left[\begin{array}{cccc} 2C_{1}^{0} & C_{2}^{0} & C_{6}^{0} & 0\\ & 2C_{3}^{0} & C_{7}^{0} & 0\\ & & 2C_{5}^{0} & 0\\ & & & 2C_{4}^{0} \end{array}\right]$$

(12) $$[C_{pq}^{k}]=\left[\begin{array}{cccc} 2C_{1}^{k}\omega_{1}^{k}+2C_{2}^{k}\omega_{2}^{k}+2C_{3}^{k}\omega_{3}^{k} & C_{13}^{k}\omega_{1}^{k}+C_{14}^{k}\omega_{2}^{k}+C_{15}^{k}\omega_{3}^{k} & C_{19}^{k}\omega_{1}^{k}+C_{20}^{k}\omega_{2}^{k}+C_{21}^{k}\omega_{3}^{k} & C_{22}^{k}\omega_{6}^{k}\\ & 2C_{4}^{k}\omega_{1}^{k}+2C_{5}^{k}\omega_{2}^{k}+2C_{6}^{k}\omega_{3}^{k} & C_{16}^{k}\omega_{1}^{k}+C_{17}^{k}\omega_{2}^{k}+C_{18}^{k}\omega_{3}^{k} & C_{23}^{k}\omega_{6}^{k}\\ & & 2C_{7}^{k}\omega_{1}^{k}+2C_{8}^{k}\omega_{2}^{k}+2C_{9}^{k}\omega_{3}^{k} & C_{24}^{k}\omega_{6}^{k}\\ & & & 2C_{10}^{k}\omega_{1}^{k}+2C_{11}^{k}\omega_{2}^{k}+2C_{12}^{k}\omega_{3}^{k} \end{array}\right]$$

(13) $$[e_{pm}^{0}]=\left[\begin{array}{c} \begin{array}{ccc} e_{1}^{0} & e_{4}^{0} & e_{7}^{0} \end{array}\\ \begin{array}{ccc} e_{2}^{0} & e_{5}^{0} & e_{8}^{0} \end{array}\\ \begin{array}{ccc} e_{3}^{0} & e_{6}^{0} & e_{9}^{0} \end{array}\\ \begin{array}{ccc} 0 & 0 & 0 \end{array} \end{array}\right]$$

(14) $$[\kappa_{mn}^{0}]=\left[\begin{array}{ccc} 2\kappa_{1}^{0} & \kappa_{4}^{0} & \kappa_{6}^{0}\\ & 2\kappa_{2}^{0} & \kappa_{5}^{0}\\ & & 2\kappa_{3}^{0} \end{array}\right]$$

(15) $$[e_{pm}^{k}]=\left[\begin{array}{ccc} e_{1}^{k}\omega_{1}^{k}+e_{2}^{k}\omega_{2}^{k}+e_{3}^{k}\omega_{3}^{k} & e_{10}^{k}\omega_{1}^{k}+e_{11}^{k}\omega_{2}^{k}+e_{12}^{k}\omega_{3}^{k} & e_{19}^{k}\omega_{1}^{k}+e_{20}^{k}\omega_{2}^{k}+e_{21}^{k}\omega_{3}^{k}\\ e_{4}^{k}\omega_{1}^{k}+e_{5}^{k}\omega_{2}^{k}+e_{6}^{k}\omega_{3}^{k} & e_{13}^{k}\omega_{1}^{k}+e_{14}^{k}\omega_{2}^{k}+e_{15}^{k}\omega_{3}^{k} & e_{22}^{k}\omega_{1}^{k}+e_{23}^{k}\omega_{2}^{k}+e_{24}^{k}\omega_{3}^{k}\\ e_{7}^{k}\omega_{1}^{k}+e_{8}^{k}\omega_{2}^{k}+e_{9}^{k}\omega_{3}^{k} & e_{16}^{k}\omega_{1}^{k}+e_{17}^{k}\omega_{2}^{k}+e_{18}^{k}\omega_{3}^{k} & e_{25}^{k}\omega_{1}^{k}+e_{26}^{k}\omega_{2}^{k}+e_{27}^{k}\omega_{3}^{k}\\ e_{28}^{k}\omega_{6}^{k} & e_{29}^{k}\omega_{6}^{k} & e_{30}^{k}\omega_{6}^{k} \end{array}\right]$$

(16) $$[\kappa_{mn}^{k}]=\left[\begin{array}{ccc} 2\kappa_{1}^{k}\omega_{1}^{k}+2\kappa_{2}^{k}\omega_{2}^{k}+2\kappa_{3}^{k}\omega_{3}^{k} & \kappa_{10}^{k}\omega_{1}^{k}+\kappa_{11}^{k}\omega_{2}^{k}+\kappa_{12}^{k}\omega_{3}^{k} & \kappa_{16}^{k}\omega_{1}^{k}+\kappa_{17}^{k}\omega_{2}^{k}+\kappa_{18}^{k}\omega_{3}^{k}\\ & 2\kappa_{4}^{k}\omega_{1}^{k}+2\kappa_{5}^{k}\omega_{2}^{k}+2\kappa_{6}^{k}\omega_{3}^{k} & \kappa_{13}^{k}\omega_{1}^{k}+\kappa_{14}^{k}\omega_{2}^{k}+\kappa_{15}^{k}\omega_{3}^{k}\\ & & 2\kappa_{7}^{k}\omega_{1}^{k}+2\kappa_{8}^{k}\omega_{2}^{k}+2\kappa_{9}^{k}\omega_{3}^{k} \end{array}\right]$$

Assuming that there is only one damage mode in the representative volume element, the relations of the strains, the stresses, the electric field intensity and the electric displacements in Equation (10) can be simplified as:

(17) $$\begin{array}{ll} \sigma_{p} & =[C_{pq}^{0}+C_{pq}^{1}]\varepsilon_{q}-[e_{pm}^{0}+e_{pm}^{1}]E_{m}\\ G_{m} & ={\left[e_{qm}^{0}+e_{qm}^{1}\right]}^{T}\varepsilon_{q}+[\kappa_{mn}^{0}+\kappa_{mn}^{1}]E_{n} \end{array}$$

where $C_{pq}^{0}$, $e_{pm}^{0}$ and $\kappa_{mn}^{0}$ are the same as before. $C_{pq}^{k}$, $e_{pm}^{k}$, $\kappa_{mn}^{k}$ are replaced by $C_{pq}^{1}$, $e_{pm}^{1}$, $\kappa_{mn}^{1}$.

In present study, consider that the matrix cracks in the piezoelectric plate are parallel to the coordinate plane 2–3, all damage variables except *ω*_1_ are zero, then the coefficient matrixes in Equations (12), (15) and (16) can be simplified as:

(18) $$[C_{pq}^{1}]=\left[\begin{array}{cccc} 2C_{1}\omega_{1} & C_{13}\omega_{1} & C_{19}\omega_{1} & 0\\ & 2C_{4}\omega_{1} & C_{16}\omega_{1} & 0\\ & & 2C_{7}\omega_{1} & 0\\ & & & 2C_{10}\omega_{1} \end{array}\right]$$

(19) $$[e_{pm}^{1}]=\left[\begin{array}{ccc} e_{1}\omega_{1} & e_{10}\omega_{1} & e_{19}\omega_{1}\\ e_{4}\omega_{1} & e_{13}\omega_{1} & e_{22}\omega_{1}\\ e_{7}\omega_{1} & e_{16}\omega_{1} & e_{25}\omega_{1}\\ 0 & 0 & 0 \end{array}\right]$$

(20) $$[\kappa_{mn}^{1}]=\left[\begin{array}{ccc} 2\kappa_{1}\omega_{1} & \kappa_{10}\omega_{1} & \kappa_{16}\omega_{1}\\ & 2\kappa_{4}\omega_{1} & \kappa_{13}\omega_{1}\\ & & 2\kappa_{7}\omega_{1} \end{array}\right]$$

Due to the fact the cracks are parallel to the coordinate plane 2 – 3, the effect of the damage on the stiffness of the plate in this coordinate plane 2 – 3 can be neglected, which means the component $C_{11}^{1}$ and $C_{11}^{3}$ of stiffness matrix due to damage effect are negligible. Then matrix (18) can be further simplified as:

(21) $$[C_{pq}^{1}]=\left[\begin{array}{cccc} 2C_{1}\omega_{1} & C_{13}\omega_{1} & C_{19}\omega_{1} & 0\\ & 0 & C_{16}\omega_{1} & 0\\ & & 0 & 0\\ & & & 2C_{10}\omega_{1} \end{array}\right]$$

Letting *σ*_3_=0 based on plane-stress assumption and using Equation (17), the constitutive relations with damage of the piezoelectric plate for the plane stress problems are obtained as follows:

(22) $$\begin{array}{lll} \sigma_{p} & =[C_{pq}]\varepsilon_{q}-[e_{pm}]E_{m}=[C_{pq}^{0}+C_{pq}^{1}]\varepsilon_{q}-[e_{pm}^{0}+e_{pm}^{1}]E_{m}\\ D_{m} & ={\left[e_{qm}\right]}^{T}\varepsilon_{q}+[\kappa_{mn}]E_{n}={\left[e_{qm}^{0}+e_{qm}^{1}\right]}^{T}\varepsilon_{q}+[\kappa_{mn}^{0}+\kappa_{mn}^{1}]E_{n} & \left(p,q=1,2,6\;m,n=1,2,3\right) \end{array}$$

where:

(23) $$[C_{pq}^{0}]=\left[\begin{array}{ccc} 2C_{1}^{0}-\frac{{\left(C_{6}^{0}\right)}^{2}}{2C_{5}^{0}} & C_{2}^{0}-\frac{C_{6}^{0}C_{7}^{0}}{2C_{5}^{0}} & 0\\ & 2C_{3}^{0}-\frac{{\left(C_{7}^{0}\right)}^{2}}{2C_{5}^{0}} & 0\\ & & 2C_{4}^{0} \end{array}\right]\underset{=}{def}\left[\begin{array}{ccc} C_{11}^{*} & C_{12}^{*} & 0\\ & C_{22}^{*} & 0\\ & & C_{66}^{*} \end{array}\right]$$

(24) $$[C_{pq}^{1}]=\left[\begin{array}{ccc} \left(2C_{1}-\frac{C_{6}^{0}C_{19}}{C_{5}^{0}}\right)\omega_{1} & \left(C_{13}-\frac{C_{7}^{0}C_{19}+C_{6}^{0}C_{16}}{2C_{5}^{0}}\right)\omega_{1} & 0\\ & -\frac{C_{7}^{0}C_{16}}{C_{5}^{0}}\omega_{1} & 0\\ & & 2C_{10}\omega_{1} \end{array}\right]\underset{=}{def}\left[\begin{array}{ccc} \alpha_{11} & \alpha_{12} & 0\\ & \alpha_{22} & 0\\ & & \alpha_{66} \end{array}\right]\omega_{1}$$

(25) $$[e_{pm}^{0}]=\left[\begin{array}{ccc} e_{1}^{0}-\frac{C_{6}^{0}e_{3}^{0}}{2C_{5}^{0}} & e_{4}^{0}-\frac{C_{6}^{0}e_{6}^{0}}{2C_{5}^{0}} & e_{7}^{0}-\frac{C_{6}^{0}e_{9}^{0}}{2C_{5}^{0}}\\ e_{2}^{0}-\frac{C_{7}^{0}e_{3}^{0}}{2C_{5}^{0}} & e_{5}^{0}-\frac{C_{7}^{0}e_{6}^{0}}{2C_{5}^{0}} & e_{8}^{0}-\frac{C_{7}^{0}e_{9}^{0}}{2C_{5}^{0}}\\ 0 & 0 & 0 \end{array}\right]\underset{=}{def}\left[\begin{array}{ccc} e_{11}^{*} & e_{12}^{*} & e_{13}^{*}\\ e_{21}^{*} & e_{22}^{*} & e_{23}^{*}\\ 0 & 0 & 0 \end{array}\right]$$

(26) $$[e_{pm}^{1}]=\left[\begin{array}{ccc} \left(e_{1}-\frac{C_{6}^{0}e_{7}+C_{19}e_{3}^{0}}{2C_{5}^{0}}\right)\omega_{1} & \left(e_{10}-\frac{C_{6}^{0}e_{16}+C_{19}e_{6}^{0}}{2C_{5}^{0}}\right)\omega_{1} & \left(e_{19}-\frac{C_{6}^{0}e_{25}+C_{19}e_{9}^{0}}{2C_{5}^{0}}\right)\omega_{1}\\ \left(e_{4}-\frac{C_{7}^{0}e_{7}+C_{16}e_{3}^{0}}{2C_{5}^{0}}\right)\omega_{1} & \left(e_{13}-\frac{C_{6}^{0}e_{16}+C_{16}e_{6}^{0}}{2C_{5}^{0}}\right)\omega_{1} & \left(e_{22}-\frac{C_{7}^{0}e_{25}+C_{16}e_{9}^{0}}{2C_{5}^{0}}\right)\omega_{1}\\ 0 & 0 & 0 \end{array}\right]\underset{=}{def}\left[\begin{array}{ccc} \beta_{11} & \beta_{12} & \beta_{13}\\ \beta_{21} & \beta_{22} & \beta_{23}\\ 0 & 0 & 0 \end{array}\right]\omega_{1}$$

(27) $$[\kappa_{mn}^{0}]=\left[\begin{array}{ccc} 2\kappa_{1}^{0}+\frac{{\left(e_{3}^{0}\right)}^{2}}{2C_{5}^{0}} & \kappa_{4}^{0}+\frac{e_{3}^{0}e_{6}^{0}}{2C_{5}^{0}} & \kappa_{6}^{0}+\frac{e_{3}^{0}e_{9}^{0}}{2C_{5}^{0}}\\ & 2\kappa_{2}^{0}+\frac{{\left(e_{6}^{0}\right)}^{2}}{2C_{5}^{0}} & \kappa_{5}^{0}+\frac{e_{6}^{0}e_{9}^{0}}{2C_{5}^{0}}\\ & & 2\kappa_{3}^{0}+\frac{{\left(e_{9}^{0}\right)}^{2}}{2C_{5}^{0}} \end{array}\right]\underset{=}{def}\left[\begin{array}{ccc} \kappa_{11}^{*} & \kappa_{12}^{*} & \kappa_{13}^{*}\\ & \kappa_{22}^{*} & \kappa_{23}^{*}\\ & & \kappa_{33}^{*} \end{array}\right]$$

(28) $$[\kappa_{mn}^{1}]=\left[\begin{array}{ccc} \left(2\kappa_{1}+\frac{e_{3}^{0}e_{7}}{C_{5}^{0}}\right)\omega_{1} & \left(\kappa_{10}+\frac{e_{3}^{0}e_{16}+e_{6}^{0}e_{7}}{2C_{5}^{0}}\right)\omega_{1} & \left(\kappa_{16}+\frac{e_{3}^{0}e_{25}+e_{9}^{0}e_{7}}{2C_{5}^{0}}\right)\omega_{1}\\ & \left(2\kappa_{4}+\frac{e_{6}^{0}e_{17}}{C_{5}^{0}}\right)\omega_{1} & \left(\kappa_{13}+\frac{e_{6}^{0}e_{25}+e_{9}^{0}e_{16}}{2C_{5}^{0}}\right)\omega_{1}\\ & & \left(2\kappa_{7}+\frac{e_{9}^{0}e_{25}}{C_{5}^{0}}\right)\omega_{1} \end{array}\right]\underset{=}{def}\left[\begin{array}{ccc} \gamma_{11} & \gamma_{12} & \gamma_{13}\\ & \gamma_{22} & \gamma_{23}\\ & & \gamma_{33} \end{array}\right]\omega_{1}$$

In the present research, the Kachanvo damage evolution law [32] is adopted for an arbitrary point *i* of the piezoelectric plate with damage:

(29) $$\frac{\partial \omega_{1}^{i}}{\partial t}=\{\begin{array}{c} B{\left(\frac{\left|\sigma_{eq}^{i}\right|}{1-\omega_{1}^{i}}-\mu \sigma_{f}\right)}^{m}\\ 0 \end{array}\begin{array}{c} \left|\sigma_{eq}^{i}\right|\geq \mu (1-\omega_{1}^{i})\sigma_{f}\\ \left|\sigma_{eq}^{i}\right|<\mu (1-\omega_{1}^{i})\sigma_{f} \end{array}$$

where *B*, *m* and *μ* are the material constants, *σ_eq_* is an equivalent stress which is based on certain failure criterion, *σ_f_* is the limit stress.

The relations between the electric fields *E_x_*, *E_y_*, *E_z_* and the electric potential *ϕ* in the Cartesian coordinate system are defined by:

(30) $$\begin{array}{lll} E_{x}=-\phi_{,x}, & E_{y}=-\phi_{,y}, & E_{z}=-\phi_{,z} \end{array}$$

For the piezoelectric plate, only thickness direction electric field *E_z_* is dominant. If the voltage applied to the piezoelectric plate with piezoelectric effect in the thickness only, then:

(31) $$E_{z}=V_{e}/h$$

where *V_e_* is the applied voltage across the thickness of piezoelectric plates.

### 2.2. Basic Equations of Piezoelectric Plates

**Figure 2.**
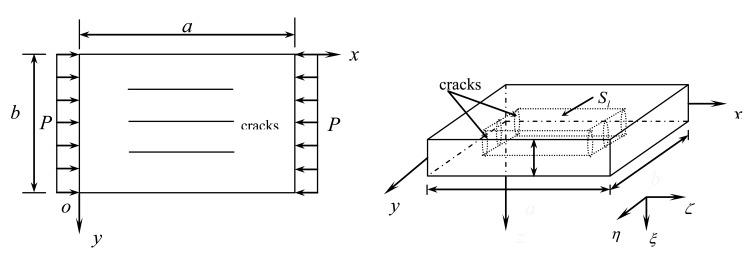
Geometric configuration of a piezoelectric plate with transverse cracks under the uniform compressive in-plane loads.

Now, consider a thin piezoelectric plate with transverse cracks subjected to uniformly distributed in-plane compressive load *P* in the *x*-direction, having thickness *h*, length *a* in the *x*-direction, width *b* in the *y*-direction shown in Figure 2. The reference surface defined by *z* = 0 is set on the middle surface of the undeformed plate.

Setting *u*, *v* and *w* as the displacement components of an arbitrary point on the mid-surface along the direction of *x*, *y* and *z*, respectively, and denote *w̅* as the initial geometric deflection. According to classical nonlinear theory, the strain components $\varepsilon_{x}^{0}$, $\varepsilon_{y}^{0}$ and $\gamma_{xy}^{0}$ of the mid-surface [33] can be written as:

(32) $$\varepsilon_{x}^{0}=u_{,x}+\frac{1}{2}{w_{,x}}^{2}+{\bar{w}}_{,x}w_{,x},\varepsilon_{y}^{0}=v_{,y}+\frac{1}{2}{w_{,y}}^{2}+{\bar{w}}_{,y}w_{,y},\gamma_{xy}^{0}=u_{,y}+v_{,x}+w_{,x}w_{,y}+{\bar{w}}_{,x}w_{,y}+{\bar{w}}_{,y}w_{,x}$$

and the curvatures *κ_x_*, *κ_y_* and *κ_xy_* of the mid-surface as:

(33) $$\begin{array}{lll} \kappa_{x}=-w_{,xx}, & \kappa_{y}=-w_{,yy}, & \kappa_{xy}=-2w_{,xy} \end{array}$$

then the nonlinear strain-displacement relations are expressed as follows:

(34) $$\begin{array}{lll} \varepsilon_{x}=\varepsilon_{x}^{0}+z\kappa_{x}, & \varepsilon_{y}=\varepsilon_{y}^{0}+z\kappa_{y}, & \gamma_{xy}=\gamma_{xy}^{0}+z\kappa_{xy} \end{array}$$

Suppose the damage variable remains constant through the thickness of plate. Denoting *N_x_*, *N_y_*, *N_xy_* as the membrane stress resultants and *M_x_*, *M_y_*, *M_xy_* as the stress couples of the plate, according to the classical nonlinear plate theory, the nonlinear governing equations of the piezoelectric plate with initial geometric deflection [33] can be written as:

(35) $$\begin{array}{l} N_{x,x}+N_{xy,y}=0\\ N_{xy,x}+N_{y,y}=0\\ M_{x,xx}+2M_{xy,xy}+M_{y,yy}+N_{x}(w_{,xx}+{\bar{w}}_{,xx})+2N_{xy}(w_{,xy}+{\bar{w}}_{,xy})+N_{y}(w_{,yy}+{\bar{w}}_{,yy})=0 \end{array}$$

Using Equations (22) and (34), the following constitutive equations can be obtained:

(36) $$\left\{\begin{array}{c} N_{x}\\ N_{y}\\ N_{xy} \end{array}\right\}=\int_{-h/2}^{h/2}\left\{\begin{array}{c} \sigma_{x}\\ \sigma_{y}\\ \sigma_{xy} \end{array}\right\}dz=\left[\begin{array}{ccc} A_{11} & A_{12} & 0\\ & A_{22} & 0\\ & & A_{66} \end{array}\right]\left\{\begin{array}{c} \varepsilon_{x}^{0}\\ \varepsilon_{y}^{0}\\ \gamma_{xy}^{0} \end{array}\right\}-\left\{\begin{array}{c} N_{x}^{p}\\ N_{y}^{p}\\ N_{xy}^{p} \end{array}\right\}$$

(37) $$\left\{\begin{array}{c} M_{x}\\ M_{y}\\ M_{xy} \end{array}\right\}=\int_{-h/2}^{h/2}z\left\{\begin{array}{c} \sigma_{x}\\ \sigma_{y}\\ \sigma_{xy} \end{array}\right\}dz=\left[\begin{array}{ccc} D_{11} & D_{12} & 0\\ & D_{22} & 0\\ & & D_{66} \end{array}\right]\left\{\begin{array}{c} \kappa_{x}\\ \kappa_{y}\\ \kappa_{xy} \end{array}\right\}-\left\{\begin{array}{c} M_{x}^{p}\\ M_{y}^{p}\\ M_{xy}^{p} \end{array}\right\}$$

where $N_{x}^{p}$, $N_{x}^{p}$, $N_{xy}^{p}$ and $M_{x}^{p}$, $M_{y}^{p}$, $M_{xy}^{p}$ represent the component resultants and couples due to the piezoelectric effect, respectively. The stiffness coefficients *A_ij_* and *D_ij_* of the piezoelectric plate are defined as follows:

(38) $$\begin{array}{lll} A_{ij}=\int_{-\overset{h}{\;}/\underset{2}{\;}}^{\overset{h}{\;}/\underset{2}{\;}}C_{ij}dz, & D_{ij}=\int_{-\overset{h}{\;}/\underset{2}{\;}}^{\overset{h}{\;}/\underset{2}{\;}}z^{2}C_{ij}dz & \left(i,j=1,2,6\right) \end{array}$$

The resultants and couples due to the piezoelectric effect can be written as:

(39) $$\begin{array}{l} \begin{array}{lll} N_{x}^{p}=\int_{-\overset{h}{\;}/\underset{2}{\;}}^{\overset{h}{\;}/\underset{2}{\;}}e_{31}\frac{V_{e}}{h}dz, & N_{y}^{p}=\int_{-\overset{h}{\;}/\underset{2}{\;}}^{\overset{h}{\;}/\underset{2}{\;}}e_{32}\frac{V_{e}}{h}dz, & N_{xy}^{p}=0 \end{array}\\ \begin{array}{lll} M_{x}^{p}=0, & M_{y}^{p}=0, & M_{xy}^{p}=0 \end{array} \end{array}$$

Introducing the following dimensionless parameters:

(40) $$\begin{array}{c} \begin{array}{llllll} \xi =\frac{x}{a}, & \eta =\frac{y}{b}, & U=\frac{u}{a}, & V=\frac{v}{b}, & W=\frac{w}{h}, & \bar{W}=\frac{\bar{w}}{h}, \end{array}\\ \begin{array}{lllllll} {\bar{A}}_{11}=\frac{A_{11}}{C_{11}^{*}h}, & {\bar{A}}_{12}=\frac{A_{12}}{C_{11}^{*}h}, & {\bar{A}}_{22}=\frac{A_{22}}{C_{11}^{*}h}, & {\bar{A}}_{66}=\frac{A_{66}}{C_{11}^{*}h}, & \lambda_{1}=\frac{h}{a}, & \lambda_{2}=\frac{h}{b}, & {\bar{D}}_{11}=\frac{D_{11}}{C_{11}^{*}h^{3}}, \end{array}\\ \begin{array}{lllllll} {\bar{D}}_{12}=\frac{D_{12}}{C_{11}^{*}h^{3}}, & {\bar{D}}_{22}=\frac{D_{22}}{C_{11}^{*}h^{3}}, & {\bar{D}}_{66}=\frac{D_{66}}{C_{11}^{*}h^{3}}, & {\bar{C}}_{12}=\frac{C_{12}}{C_{11}^{*}}, & {\bar{C}}_{11}=\frac{C_{11}}{C_{11}^{*}}, & {\bar{\sigma }}_{eq}=\frac{\sigma_{eq}}{C_{11}^{*}}, & {\bar{\sigma }}_{f}=\frac{\sigma_{f}}{C_{11}^{*}}, \end{array}\\ \begin{array}{llllllll} {\bar{\alpha }}_{11}=\frac{\alpha_{11}}{C_{11}^{*}}, & {\bar{\alpha }}_{12}=\frac{\alpha_{12}}{C_{11}^{*}}, & {\bar{\alpha }}_{22}=\frac{\alpha_{22}}{C_{11}^{*}}, & {\bar{\alpha }}_{66}=\frac{\alpha_{66}}{C_{11}^{*}}, & \bar{e_{31}}=\frac{e_{31}}{e_{31}^{*}}, & \bar{e_{32}}=\frac{e_{32}}{e_{31}^{*}}, & {\bar{\beta }}_{31}=\frac{\beta_{31}}{e_{31}^{*}}, & {\bar{\beta }}_{32}=\frac{\beta_{32}}{e_{31}^{*}}, \end{array}\\ \begin{array}{llll} \bar{z}=\frac{z}{h}, & \tau =\frac{t}{h}\sqrt{\frac{C_{11}^{*}}{\rho }}, & \bar{P}=\frac{P}{C_{11}^{*}}, & \bar{V_{e}}=\frac{e_{31}^{*}V_{e}}{C_{11}^{*}h} \end{array} \end{array}$$

By using Equations (22), (32–39) and (40), the dimensionless nonlinear governing equations of piezoelectric plate with initial geometric deflection under compressive loads in-plane including damage effects are obtained and expressed in terms of *U*,*V* and *W* as follow:

(41) $$\begin{array}{l} {\bar{A}}_{11,\xi }(U_{,\xi }+\frac{1}{2}{\lambda_{1}}^{2}{W_{,\xi }}^{2}+{\lambda_{1}}^{2}{\bar{W}}_{,\xi }W_{,\xi })+{\bar{A}}_{11}(U_{,\xi \xi }+{\lambda_{1}}^{2}W_{,\xi }W_{,\xi \xi }+{\lambda_{1}}^{2}{\bar{W}}_{,\xi \xi }W_{,\xi }+{\lambda_{1}}^{2}{\bar{W}}_{,\xi }W_{,\xi \xi })+{\bar{A}}_{12,\xi }(V_{,\eta }\\ +\frac{1}{2}{\lambda_{2}}^{2}{W_{,\eta }}^{2}+{\lambda_{2}}^{2}{\bar{W}}_{,\eta }W_{,\eta })+{\bar{A}}_{12}(V_{,\xi \eta }+{\lambda_{2}}^{2}W_{,\eta }W_{,\xi \eta }+{\lambda_{2}}^{2}{\bar{W}}_{,\xi \eta }W_{,\eta }+{\lambda_{2}}^{2}{\bar{W}}_{,\eta }W_{,\xi \eta })+\frac{\lambda_{2}}{\lambda_{1}}{\bar{A}}_{66,\eta }(\frac{\lambda_{2}}{\lambda_{1}}U_{,\eta }+\\ \frac{\lambda_{1}}{\lambda_{2}}V_{,\xi }+\lambda_{1}\lambda_{2}W_{,\xi }W_{,\eta }+\lambda_{1}\lambda_{2}{\bar{W}}_{,\xi }W_{,\eta }+\lambda_{1}\lambda_{2}W_{,\xi }{\bar{W}}_{,\eta })+{\bar{A}}_{66}(\frac{\lambda_{2}^{2}}{\lambda_{1}^{2}}U_{,\eta \eta }+V_{,\xi \eta }+{\lambda_{2}}^{2}W_{,\eta }W_{,\xi \eta }+{\lambda_{2}}^{2}W_{,\xi }W_{,\eta \eta }+\\ {\lambda_{2}}^{2}{\bar{W}}_{,\xi \eta }W_{,\eta }+{\lambda_{2}}^{2}{\bar{W}}_{,\eta \eta }W_{,\xi }+{\lambda_{2}}^{2}{\bar{W}}_{,\xi }W_{,\eta \eta }+{\lambda_{2}}^{2}{\bar{W}}_{,\eta }W_{,\xi \eta })-{\bar{e_{31}}}_{,\xi }\bar{V_{e}}=0\\ {\bar{A}}_{12,\eta }(U_{,\xi }+\frac{1}{2}{\lambda_{1}}^{2}{W_{,\xi }}^{2}+{\lambda_{1}}^{2}{\bar{W}}_{,\xi }W_{,\xi })+{\bar{A}}_{12}(U_{,\xi \eta }+{\lambda_{1}}^{2}W_{,\xi }W_{,\xi \eta }+{\lambda_{1}}^{2}{\bar{W}}_{,\xi \eta }W_{,\xi }+{\lambda_{1}}^{2}{\bar{W}}_{,\xi }W_{,\xi \eta })+{\bar{A}}_{22,\eta }(V_{,\eta }\\ +\frac{1}{2}{\lambda_{2}}^{2}{W_{,\eta }}^{2}+{\lambda_{2}}^{2}{\bar{W}}_{,\eta }W_{,\eta })+{\bar{A}}_{22}(V_{,\eta \eta }+{\lambda_{2}}^{2}W_{,\eta }W_{,\eta \eta }+{\lambda_{2}}^{2}W_{,\eta }{\bar{W}}_{,\eta \eta }+{\lambda_{2}}^{2}{\bar{W}}_{,\eta }W_{,\eta \eta })+\frac{\lambda_{1}}{\lambda_{2}}{\bar{A}}_{66,\xi }(\frac{\lambda_{2}}{\lambda_{1}}U_{,\eta }+\\ \frac{\lambda_{1}}{\lambda_{2}}V_{,\xi }+\lambda_{1}\lambda_{2}W_{,\xi }W_{,\eta }+\lambda_{1}\lambda_{2}{\bar{W}}_{,\xi }W_{,\eta }+\lambda_{1}\lambda_{2}W_{,\xi }{\bar{W}}_{,\eta })+{\bar{A}}_{66}(U_{,\xi \eta }+\frac{\lambda_{1}^{2}}{\lambda_{2}^{2}}V_{,\xi \xi }+{\lambda_{1}}^{2}W_{,\eta }W_{\xi \xi }+{\lambda_{1}}^{2}W_{,\xi }W_{,\xi \eta }+\\ {\lambda_{1}}^{2}{\bar{W}}_{,\xi \xi }W_{,\eta }+{\lambda_{1}}^{2}{\bar{W}}_{,\xi }W_{,\xi \eta }+{\lambda_{1}}^{2}{\bar{W}}_{,\xi \eta }W_{,\xi }+{\lambda_{1}}^{2}{\bar{W}}_{,\eta }W_{,\xi \xi })-{\bar{e_{32}}}_{,\eta }\bar{V_{e}}=0\\ -{\lambda_{1}}^{4}({\bar{D}}_{11,\xi \xi }W_{,\xi \xi }+2{\bar{D}}_{11,\xi }W_{,\xi \xi \xi }+{\bar{D}}_{11}W_{,\xi \xi \xi \xi })-{\lambda_{1}}^{2}{\lambda_{2}}^{2}({\bar{D}}_{12,\xi \xi }W_{\eta \eta }+2{\bar{D}}_{12,\xi }W_{,\xi \eta \eta }+{\bar{D}}_{12}W_{,\xi \xi \eta \eta })-4{\lambda_{1}}^{2}{\lambda_{2}}^{2}(\\ {\bar{D}}_{66,\xi \eta }W_{,\xi \eta }+{\bar{D}}_{66,\xi }W_{,\xi \eta \eta }+{\bar{D}}_{66,\eta }W_{,\xi \xi \eta }+{\bar{D}}_{66}W_{,\xi \xi \eta \eta })-{\lambda_{1}}^{2}{\lambda_{2}}^{2}({\bar{D}}_{12,\eta \eta }W_{,\xi \xi }+2{\bar{D}}_{12,\eta }W_{\xi \xi \eta }+{\bar{D}}_{12}W_{,\xi \xi \eta \eta })-\\ {\lambda_{2}}^{4}({\bar{D}}_{22,\eta \eta }W_{,\eta \eta }+2{\bar{D}}_{22,\eta }W_{,\eta \eta \eta }+{\bar{D}}_{22}W_{,\eta \eta \eta \eta })+{\lambda_{1}}^{2}[{\bar{A}}_{11}(U_{,\xi }+\frac{1}{2}{\lambda_{1}}^{2}{W_{,\xi }}^{2}+{\lambda_{1}}^{2}{\bar{W}}_{,\xi }W_{,\xi })+{\bar{A}}_{12}(V_{,\eta }+\frac{1}{2}{\lambda_{2}}^{2}{W_{,\eta }}^{2}\\ +{\lambda_{2}}^{2}{\bar{W}}_{,\eta }W_{,\eta })-\bar{e_{31}}\bar{V_{e}}](W_{,\xi \xi }+{\bar{W}}_{,\xi \xi })+2\lambda_{1}\lambda_{2}{\bar{A}}_{66}(\frac{\lambda_{2}}{\lambda_{1}}U_{,\eta }+\frac{\lambda_{1}}{\lambda_{2}}V_{,\xi }+\lambda_{1}\lambda_{2}W_{,\xi }W_{,\eta }+\lambda_{1}\lambda_{2}{\bar{W}}_{,\xi }W_{,\eta }+\lambda_{1}\lambda_{2}W_{,\xi }{\bar{W}}_{,\eta })(W_{,\xi \eta }\\ +{\bar{W}}_{,\xi \eta })+{\lambda_{2}}^{2}[{\bar{A}}_{12}(U_{,\xi }+\frac{1}{2}{\lambda_{1}}^{2}{W_{,\xi }}^{2}+{\lambda_{1}}^{2}{\bar{W}}_{,\xi }W_{,\xi })+{\bar{A}}_{22}(V_{,\eta }+\frac{1}{2}{\lambda_{2}}^{2}{W_{,\eta }}^{2}+{\lambda_{2}}^{2}{\bar{W}}_{,\eta }W_{,\eta })-\bar{e_{32}}\bar{V_{e}}](W_{,\eta \eta }+{\bar{W}}_{,\eta \eta })=0 \end{array}$$

Suppose the boundary of the piezoelectric plate is simply movably supported, the dimensionless boundary conditions can be expressed as:

(42) $$\begin{array}{c} \begin{array}{lll} \xi =0,1: & V=0, & {\bar{A}}_{11}(U{,}_{\xi }+\frac{1}{2}{\lambda_{1}}^{2}{W_{,\xi }}^{2}+{\lambda_{1}}^{2}\bar{W}{,}_{\xi }\bar{W}{,}_{\xi })-\bar{e_{31}^{1}}\bar{V_{e}}=-\bar{p} \end{array}\\ \begin{array}{ll} W=0, & W_{,\xi \xi }=0 \end{array}\\ \begin{array}{lll} \eta =0.1: & U=0, & {\bar{A}}_{22}(V_{,\eta }+\frac{1}{2}{\lambda_{2}}^{2}{W_{,\eta }}^{2}+{\lambda_{2}}^{2}{\bar{W}}_{,\eta }W_{,\eta })-\bar{e_{32}^{1}}\bar{V_{e}}=0 \end{array}\\ \begin{array}{ll} W=0, & W_{,\eta \eta }=0 \end{array} \end{array}$$

The dimensionless damage evolution equation of the piezoelectric plate subjected to the uniformly in-plane compressive load can be written respectively as follows:

(43) $$\frac{\partial \omega_{1}^{i}}{\partial \tau }=\{\begin{array}{c} \bar{B}{\left(\frac{\left|{\bar{\sigma }}_{eq}^{i}\right|}{1-\omega_{1}^{i}}-\mu {\bar{\sigma }}_{f}\right)}^{m}\\ 0 \end{array}\begin{array}{c} \left|{\bar{\sigma }}_{eq}^{i}\right|\geq \mu (1-\omega_{1}^{i}){\bar{\sigma }}_{f}\\ \left|{\bar{\sigma }}_{eq}^{i}\right|<\mu (1-\omega_{1}^{i}){\bar{\sigma }}_{f} \end{array}$$

Taking the mid-surface normal stress of the piezoelectric plate as the equivalent stress *σ̅_eq_* that is parallel to the fibrous direction, it can be presented as:

(44) $${\bar{\sigma }}_{eq}={\bar{C}}_{11}(U_{,\xi }+\frac{1}{2}{\lambda_{1}}^{2}{W_{,\xi }}^{2}+{\lambda_{1}}^{2}\bar{W_{,\xi }}W_{,\xi })+{\bar{C}}_{12}(V_{,\eta }+\frac{1}{2}{\lambda_{2}}^{2}{W_{,\eta }}^{2}+{\lambda_{2}}^{2}\bar{W_{,\eta }}W_{,\eta })-\bar{e_{31}^{1}}\bar{V_{e}}$$

## 3. Solution Methodology

Suppose the dimensionless initial geometric deflection is taken as:

(45) $$\bar{W}=\bar{W_{0}}\sin p\pi \xi \sin q\pi \eta$$

where *p* and *q* are the mode number in the *ξ*-direction and *η*-direction of the piezoelectric plate, respectively. Since the load and the structure are symmetric, only one quarter of the plate needs be considered. So the domain of the problem is selected as 0 ≤ *ξ* ≤ 1/2, 0 ≤ *η* ≤ 1/2.

To seek the approximate solutions of the governing Equations (41) which satisfied the boundary conditions (42), the unknown functions *U*,*V* and *W* are separated both for space and for time. The finite difference method is used for space, and the partial derivatives with respect to the space coordinate variables are replaced by difference form. The time *τ* is equally divided into small time segments Δ*τ*, and the whole equations are iterated to seek solutions. At each step of the iteration, the nonlinear items in the equations and the boundary conditions are linearized. For example, at the step *J*, the nonlinear items may be transformed to:

(46) $${\left(x\cdot y\right)}_{J}={\left(x\right)}_{J}\cdot {\left(y\right)}_{J_{p}}$$

where (*y*)*_J_p__* is the average value of those obtained in the preceding two iterations. For the initial step of the iteration, it can be determined by using the quadratic extrapolation, *i.e.*:

(47) $${\left(y\right)}_{J_{p}}=A{\left(y\right)}_{J-1}+B{\left(y\right)}_{J-2}+C{\left(y\right)}_{J-3}$$

and for the different step of the iteration, the coefficients *A*, *B* and *C* can be expressed as follows:

(48) $$\begin{array}{ll} J=1: & A=1,B=0,C=0\\ J=2: & A=2,B=-1,C=0\\ J\geq 3: & A=3,B=-3,C=1 \end{array}$$

Moreover, using the Newmark scheme, the inertia in Equation (41) can be expressed as follows:

(49) $$\begin{array}{l} {\left(W_{,\tau \tau }\right)}_{J}=\frac{4(W_{J}-W_{J-1})}{{\left(\Delta \tau \right)}^{2}}-\frac{4{\left(W_{,\tau }\right)}_{J-1}}{\Delta \tau }-{\left(W_{,\tau \tau }\right)}_{J-1}\\ {\left(W_{,\tau }\right)}_{J}={\left(W_{,\tau }\right)}_{J-1}+\frac{1}{2}[{\left(W_{,\tau \tau }\right)}_{J-1}+{\left(W_{,\tau \tau }\right)}_{J}]\Delta \tau \\ W_{J}=W_{J-1}+{\left(W_{,\tau }\right)}_{J-1}\Delta \tau +\frac{1}{4}[{\left(W_{,\tau \tau }\right)}_{J-1}+{\left(W_{,\tau \tau }\right)}_{J}]{\left(\Delta \tau \right)}^{2} \end{array}$$

For every time step, the iteration lasts until the difference of the present value and the former is smaller than 0.1%, then continue the calculation of the next step.

## 4. Numerical Results

### 4.1. Comparison Study

**Figure 3.**
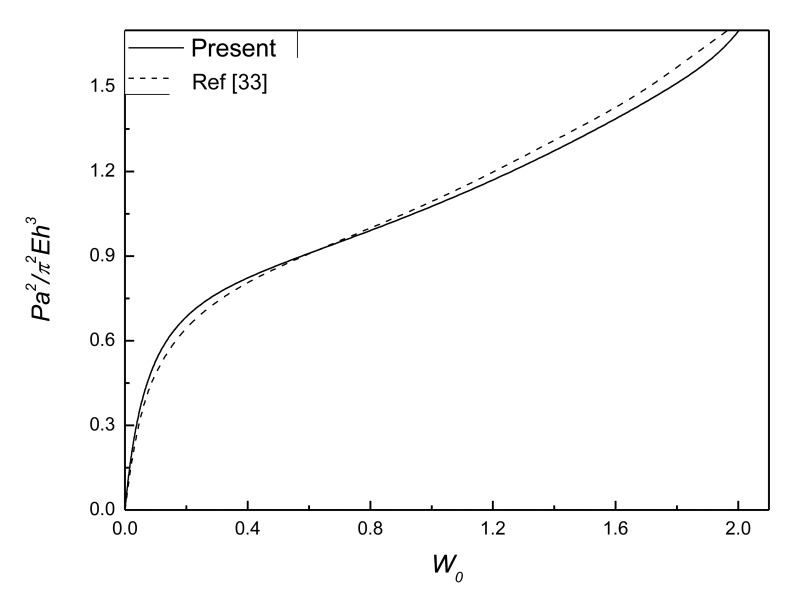
Comparison of postbuckling response curves for isotropic rectangular plate with initial deflection (*W̅*_0_ =0.1, *v* = 1/3).

To ensure the accuracy and effectiveness of the present method, a test example was calculated for postbuckling analysis of isotropic rectangular plate with initial geometric deflection. Comparison of postbuckling response curves for isotropic rectangular plate with initial geometric deflection is shown in Figure 3. The boundaries of the plate are clamped movable edges. *W*_0_ denotes the center deflection of the plate. The close agreements between the present results and those of reference [33] demonstrate the present method is accurate and effective.

### 4.2. Parametric Study

To study the piezo-effects and damage effects on the postbuckling behavior of the plates, several numerical examples were solved for initial flat and deflected plate. A piezoelectric plate consisting of the PZT-5A including initial damage is considered for postbuckling analysis. The material properties of PZT-5A are given as follows:

(50) $$\begin{array}{l} E_{11}=E_{22}=61.0\text{GPa},E_{33}=53.2\text{GPa},\upsilon_{12}=0.35,\upsilon_{13}=\upsilon_{23}=0.38\\ G_{12}=22.6\text{GPa},G_{13}=G_{23}=21.1\text{GPa},\rho =7.75\times 10^{3}(\text{kg}/{\text{m}}^{3})\\ e_{31}=e_{32}=7.209\text{C}/{\text{m}}^{2},e_{33}=15.118\text{C}/{\text{m}}^{2},e_{15}=e_{24}=12.322\text{C}/{\text{m}}^{2}\\ \kappa_{11}=\kappa_{22}=1.53\times 10^{-8}\text{F}/\text{m},\kappa_{33}=1.5\times 10^{-8}\text{F}/\text{m} \end{array}$$

When the effect of damage is omitted and the linear strain-displacement relations are adopted, the dimensionless governing equation corresponding to Equation (41) is presented as:

(51) $$\begin{array}{l} -{\lambda_{1}}^{4}{\bar{D}}_{11}W_{,\xi \xi \xi \xi }-2{\lambda_{1}}^{2}{\lambda_{2}}^{2}{\bar{D}}_{12}W_{,\xi \xi \eta \eta }-4{\lambda_{1}}^{2}{\lambda_{2}}^{2}{\bar{D}}_{66}W_{,\xi \xi \eta \eta }-{\lambda_{2}}^{4}{\bar{D}}_{22}W_{,\eta \eta \eta \eta }\\ -{\lambda_{1}}^{2}(\bar{P}+\bar{e_{31}}{\bar{V}}_{e})(W_{,\xi \xi }+{\bar{W}}_{,\xi \xi })=0 \end{array}$$

The corresponding dimensionless boundary conditions of the simply movable supported plate can be written as:

(52) $$\begin{array}{llllll} \xi & =0,1: & W & =0, & W_{,\xi \xi } & =0\\ \eta & =0,1: & W & =0, & W_{,\eta \eta } & =0 \end{array}$$

Considering a harmonic displacement solution for this buckling problem (51), the displacement that satisfies with the boundary conditions (52) can be expressed as:

(53) $$W(\xi ,\eta )=W_{0}\sin m\pi \xi \sin n\pi \eta$$

Substituting Equation (53) into the Equation (51), the buckling load *P̅_mn_* of a perfect plate and the relation of the center deflection and the compressive load in-plane of the plate with initial geometric imperfection can be obtained, respectively, as:

(54) $${\bar{P}}_{mn}=\pi^{2}({\lambda_{1}}^{2}m^{2}{\bar{D}}_{11}+2{\lambda_{2}}^{2}n^{2}{\bar{D}}_{12}+\frac{{\lambda_{2}}^{4}n^{4}}{{\lambda_{1}}^{2}m^{2}}{\bar{D}}_{22}+4{\lambda_{2}}^{2}n^{2}{\bar{D}}_{66})-\bar{e_{31}}{\bar{V}}_{e}$$

(55) $$W_{0}(\bar{P})=\frac{(P_{mn}+\bar{e_{31}}{\bar{V}}_{e})\bar{W_{0}}}{P_{mn}-\bar{P}}$$

The least critical buckling load *P̅_cr_* of the piezoelectric plate is determined by applying Equation (54) for the buckling mode (*m*, *n*) = (1, 1) (*m*, *n*) =(1, 1), as:

(56) $${\bar{P}}_{cr}=\pi^{2}({\lambda_{1}}^{2}{\bar{D}}_{11}+2{\lambda_{2}}^{2}{\bar{D}}_{12}+\frac{{\lambda_{2}}^{4}}{{\lambda_{1}}^{2}}{\bar{D}}_{22}+4{\lambda_{2}}^{2}{\bar{D}}_{66})-\bar{e_{31}}{\bar{V}}_{e})$$

**Figure 4.**
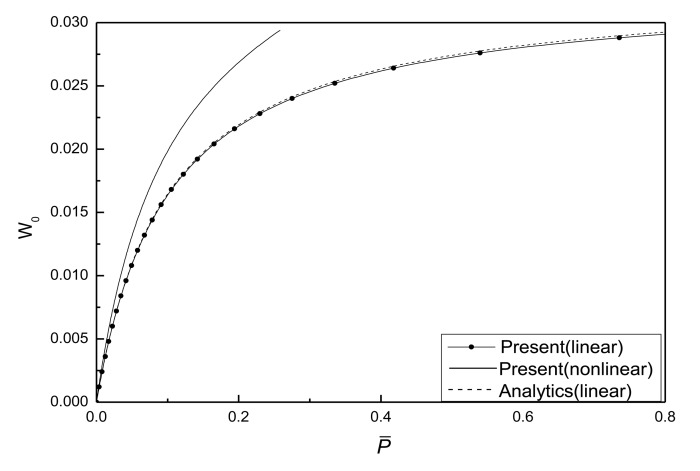
Response curves of the centre deflection *vs*. compressive loads in plane without damage.

When the geometric parameters are given as *λ*_1_ = *λ*_2_ =0.1, *W̅*_0_ =0.1, the critical buckling load *P̅_cr_* is obtained as 0.03290 and 0.03272 by using the Equation (56) and the algorithm in the present paper, respectively. Figure 4 shows the relations of the center deflection *W*_0_ of the piezoelectric plate and the in-plane compressive load *P̅* without considering the damage effect. It can be seen that the nonlinearity of the plate has great influence on the postbuckling paths of the piezoelectric plate with initial deflection.

**Figure 5.**
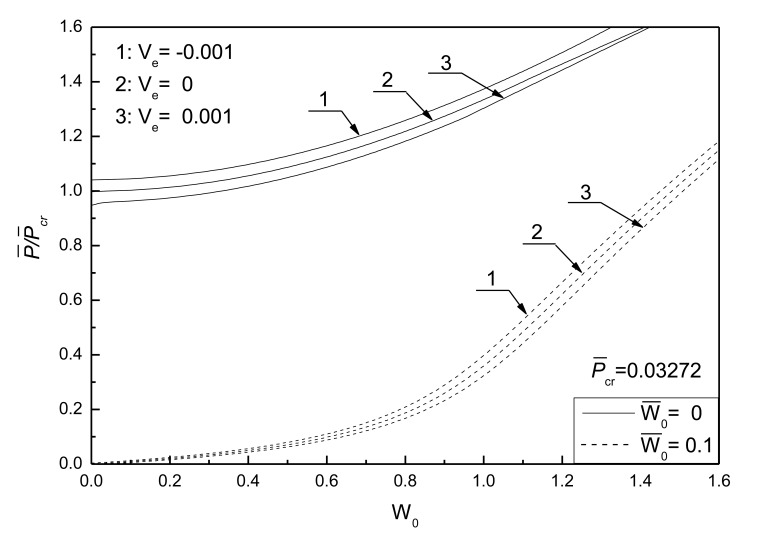
Comparisons of postbuckling response curves for initially flat and deflected piezoelectric plate without damage under different electrical loads.

**Figure 6.**
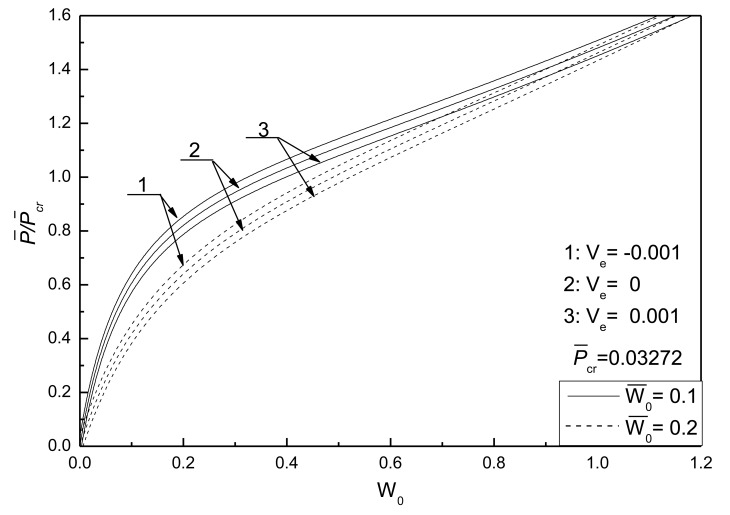
Effect of electrical loads on postbuckling response curves of piezoelectric plate without damage under two different initial deflections.

**Figure 7.**
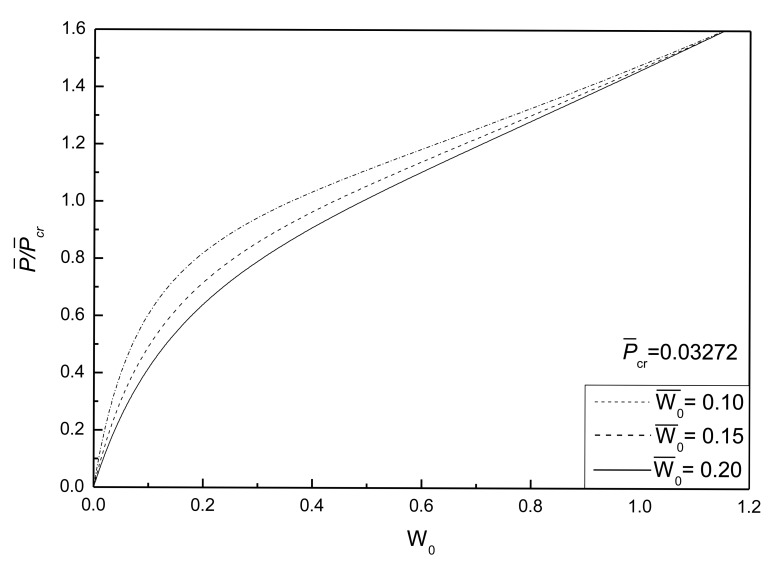
Effect of initial geometric deflections on the postbuckling response curves of piezoelectric plate without damage.

**Figure 8.**
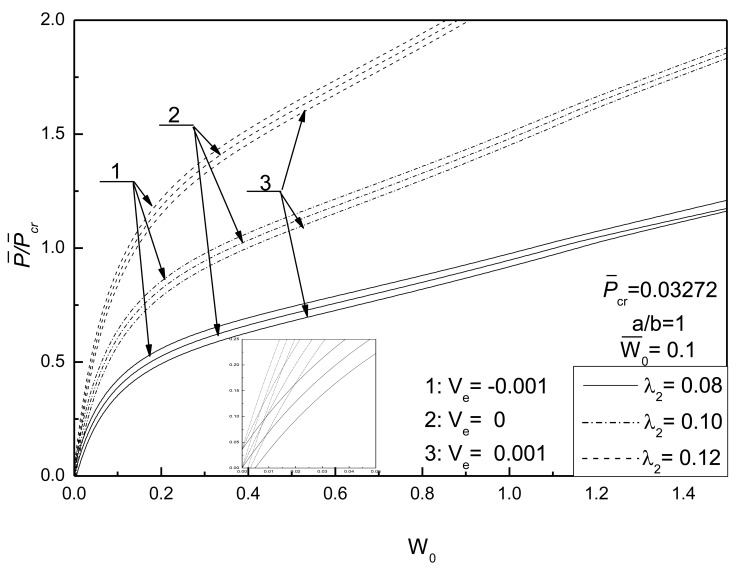
Effect of thick-span ratio on postbuckling response curves of piezoelectric plate without damage under different electrical loads (the inset figure is a zoom-in snapshot of the region around orgin point to depict the difference of three cases).

**Figure 9.**
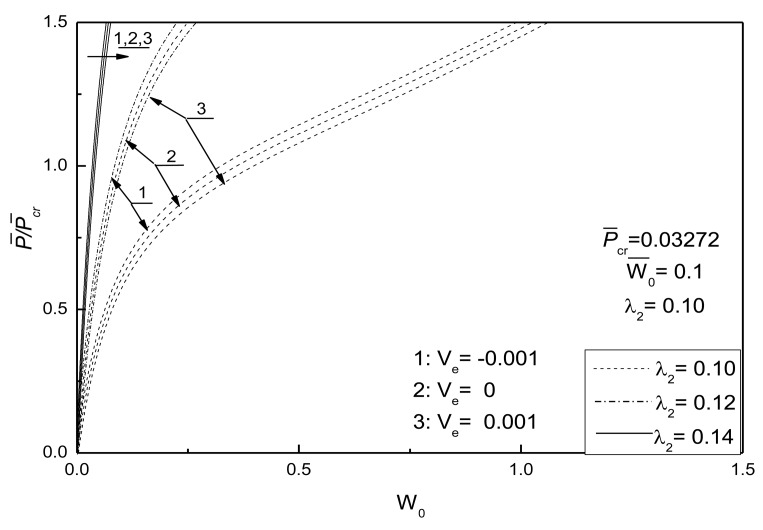
Effect of aspect ratio on postbuckling response curves of piezoelectric plate without damage under different electrical loads.

**Figure 10.**
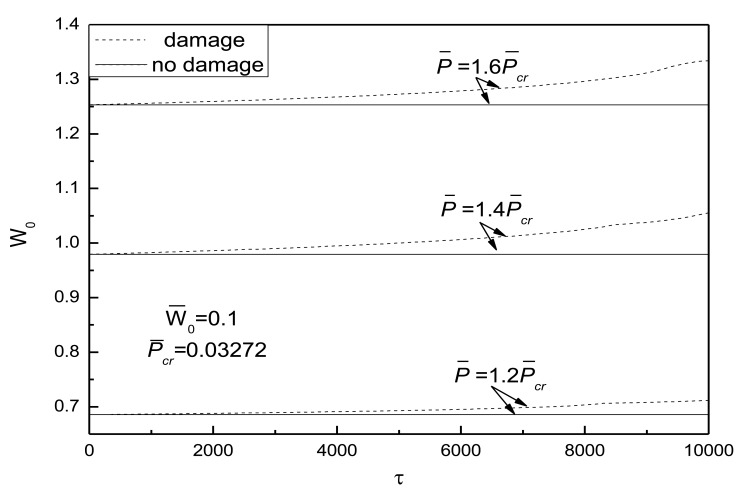
Effect of external loads on postbuckling response curves of piezoelectric plate with damage and initial deflection.

**Figure 11.**
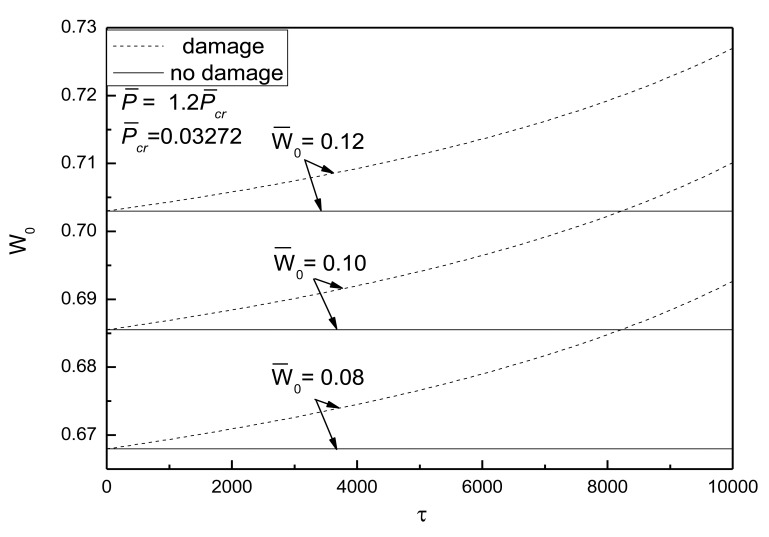
Effect of initial deflections on the postbuckling response curves of piezoelectric plate with damage.

**Figure 12.**
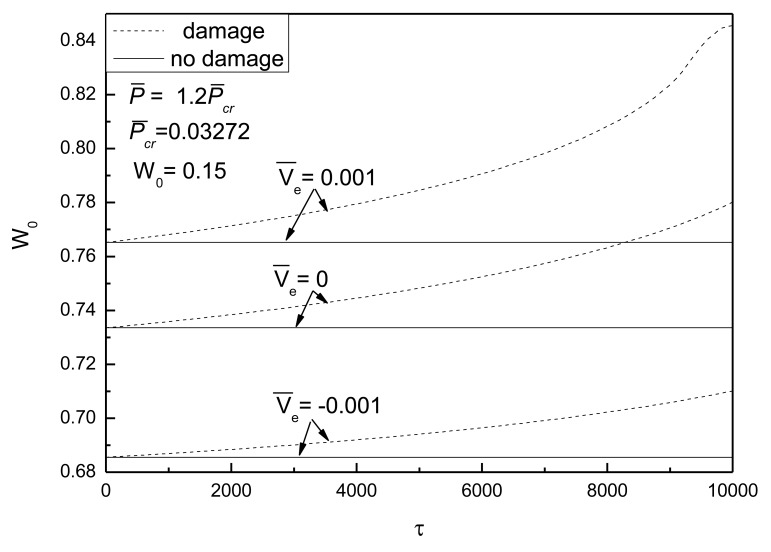
Effect of electrical loads on postbuckling response curves of piezoelectric plate with damage and initial deflection.

**Figure 13.**
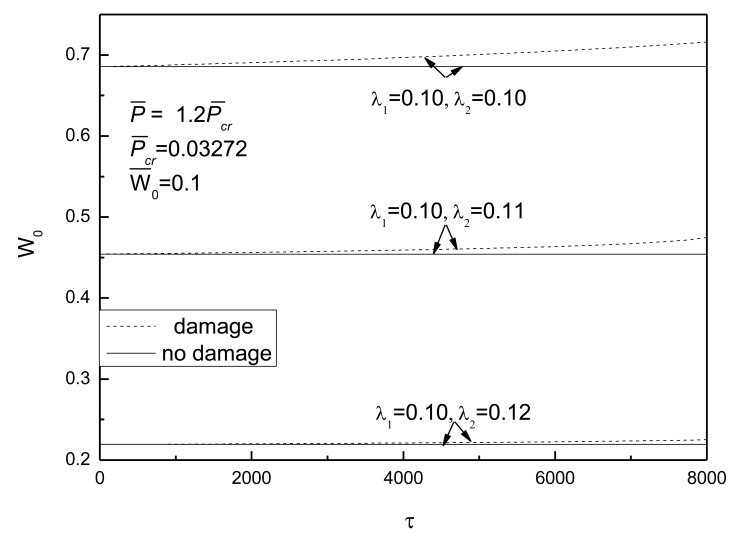
Effect of aspect ratio on postbuckling response curves of piezoelectric plate with damage and initial deflection.

**Figure 14.**
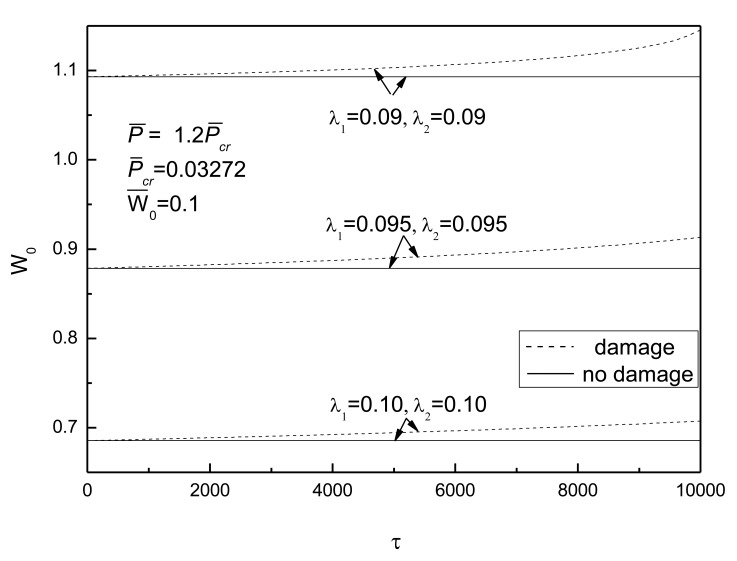
Effect of thick-span ratio on postbuckling response curves of piezoelectric plate with damage and initial deflection.

A parametric study has been carried out and typical results are shown in Figures 5, 6, 7, 8, 9, 10, 11, 12, 13 and 14. It should be appreciated that in all figures *W*_0_, *W̅*, *V̅_e_* and *P̅_cr_* denote the dimensionless maximum deflection, the dimensionless maximum initial deflection of the plate, the dimensionless applied voltage acted upon the plate and the dimensionless least critical buckling load. In all examples the least critical buckling load is taken as *P̅_cr_* =0.03272, which was calculated without considering piezo-effects and with the geometric parameters *λ*_1_ = *λ*_2_ = 0.01.

When the damage effect is in consideration, the material parameters related to damage in all examples are taken as:

*B̅* = 0.5, *n* = 1.1, *σ̅_f_* = 4×10^−2^, $\bar{\alpha_{11}}=-0.1$, $\bar{\alpha_{12}}=-0.04$, $\bar{\alpha_{22}}=-0.1$, $\bar{\alpha_{66}}=-0.04$, $\bar{\beta_{31}}=0.1$, $\bar{\beta_{32}}=-0.1$, *μ* = 0.06.

Figure 5 shows the postbuckling response curves for an initially flat and deflected piezoelectric plate without damage under different electrical loads and Figure 6 shows the effects of electrical loads on the postbuckling response curve of a piezoelectric plate without damage under two initial deflection conditions, respectively. The geometric parameters are given as *λ*_1_ = *λ*_2_ = 0.1. Three electrical load conditions, referred as 1, 2 and 3, are considered. It can be seen that the negative control voltage results in the increase of the buckling load and the decrease of postbuckled deflection at the same compressive loads. In contrast, the positive control voltage decreases the buckling load and induces larger postbuckled deflections. It can be concluded that the positive control voltage acting upon the piezoelectric plate is equivalent to a compressive piezoelectric force acting in the in-plane direction of the plate to some certain extent, which leads to the smaller buckling loads.

Figure 7 shows the effect of initial deflections on the postbuckling response curve of piezoelectric plate without damage. The geometric parameters are given as *λ*_1_ = *λ*_2_ = 0.1. It can be seen that the larger the initial deflections of the plate, the larger the postbuckled deflection of the plate under the same compressive load, and that the postbuckled deflections of the plate under different initial deflections will reach the same value with the increase of postbuckling loads.

Figure 8 shows the effect of thick-span ratio of the plate on postbuckling response curves of piezoelectric plate without damage under different electrical loads. It can be found that the buckling loads increase with the increase of the thick-span ratio of plate, and that the control voltage has a small effect on the postbuckling behaviors of the plate with lower thick-span ratio.

Figure 9 shows the effect of aspect ratio of the plate on postbuckling response curves of piezoelectric plate without damage under different electrical loads. As expected, these results show that the buckling loads are increased by increasing the aspect ratio of the plate. It can also be found that the effect of control voltage is more pronounced for the square plate than for the rectangular plate.

Figure 10 shows the effect of external loads on postbuckling response curves of piezoelectric plate with damage and initial deflection. The electrical load is taken as *V̅_e_* =0. It can be concluded that the larger the external compressive loads, the quicker the development of damage and the more obvious the effect of damage on the postbuckling deflection. From Figure 11, the conclusion that the change of initial deflection has a small effect on the postbuckled deflection and the damage effect varies slightly can be drawn.

Figure 12 shows the effect of electrical loads on the postbuckling response curves of piezoelectric plate with damage and initial deflection. It can be seen that the control voltage has a notable effect on the postbuckled deflection and the effect of damage varies greatly. The negative control voltage results in a smaller rate of damage development of the plate than that of the same plate without electrical loads, so it can be concluded that the positive control voltage can increase the rate of degradation of the stiffness of the piezoelectric plate. This can be explained as follows: the effect of positive control voltage on the damaged piezoelectric plate is equivalent to a tensile force acting in the in-plane direction of the plate, resulting in the acceleration of the piezoelectric plate degradation process. Therefore, it demonstrates a prominent effect on the deformation of piezoelectric plate with damage under the same loading capacity.

Figure 13 shows the effect of aspect ratio on postbuckling response curves of the piezoelectric plate with damage and initial deflections and Figure 14 shows the effect of thick-span ratio on postbuckling response curves of the piezoelectric plate with damage and initial deflections. The electrical load is taken as *V̅_e_* =0. From Figure 13, it can be seen that the bigger the aspect ratio of the plate, the smaller the postbuckled deflection of the plate and the smaller the effect of damage. Figure 14 indicates that the larger the thick-span ratio, the smaller the postbuckled deflection of the plate and the smaller the effect of damage.

## 5. Conclusions

This paper presents an approach to investigate the postbuckling analysis of piezoelectric plates including damage effects using Talreja's tensor valued internal state damage variables and the Kachanvo damage evolution equation. The effects of applied voltage, plate aspect ratio, thick-span ratio, damage as well as initial geometric deflections on the postbuckling behaviors of the piezoelectric plate are investigated. Numerical results show that the nonlinearity of structure has a great influence on the postbuckling paths of the piezoelectric plate. The negative control voltage results in the increase of the buckling loads and the decrease of postbuckled deflections under the same in-plane compressive loads, whereas the positive control voltage decreases the buckling loads and induces larger postbuckled deflections. The buckling loads increase with the increase of the thick-span ratio of the plate, and the control voltage has a small effect on the postbuckling behaviors of the plate with lower thick-span ratio. When the damage and damage evolution are considered, the postbuckled deflection of the plate will gradually grow with the increase of the time until the damage reaches a characteristic damage state. The external in-plane compressive loads and the applied control voltage have great effects on the postbuckled deflections of the plate and the damage development. The negative control voltage can decrease the degradation rate of the stiffness of the piezoelectric structures and will provide a control mean for the damaged smart structures.
